# Clinical Impact of MALDI-TOF MS Identification and Rapid Susceptibility Testing on Adequate Antimicrobial Treatment in Sepsis with Positive Blood Cultures

**DOI:** 10.1371/journal.pone.0156299

**Published:** 2016-05-26

**Authors:** Alexia Verroken, Lydwine Defourny, Olivier le Polain de Waroux, Leïla Belkhir, Pierre-François Laterre, Michel Delmée, Youri Glupczynski

**Affiliations:** 1 Institut de recherche expérimentale et clinique (IREC), pôle de microbiologie (MBLG), Université catholique de Louvain, Brussels, Belgium; 2 Laboratoire de microbiologie, Cliniques universitaires Saint-Luc – Université catholique de Louvain, Brussels, Belgium; 3 Department of Infectious Disease Epidemiology, London School of Hygiene and Tropical Medicine, London, United Kingdom; 4 Département de médecine interne et pathologies infectieuses, Cliniques universitaires Saint-Luc – Université catholique de Louvain, Brussels, Belgium; 5 Département des soins intensifs, Cliniques universitaires Saint-Luc – Université catholique de Louvain, Brussels, Belgium; 6 National Reference Centre for Monitoring Antimicrobial Resistance in Gram-negative bacteria, CHU UCL Namur, Yvoir, Belgium; Harvard Medical School, UNITED STATES

## Abstract

Shortening the turn-around time (TAT) of positive blood culture (BC) identification (ID) and susceptibility results is essential to optimize antimicrobial treatment in patients with sepsis. We aimed to evaluate the impact on antimicrobial prescription of a modified workflow of positive BCs providing ID and partial susceptibility results for Enterobacteriaceae (EB), *Pseudomonas aeruginosa* and *Staphylococcus aureus* on the day of BC positivity detection. This study was divided into a pre-intervention period (P0) with a standard BC workflow followed by 2 intervention periods (P1, P2) with an identical modified workflow. ID was performed with MALDI-TOF MS from blood, on early or on overnight subcultures. According to ID results, rapid phenotypic assays were realized to detect third generation cephalosporin resistant EB/*P*. *aeruginosa* or methicillin resistant *S*. *aureus*. Results were transmitted to the antimicrobial stewardship team for patient’s treatment revision. Times to ID, to susceptibility results and to optimal antimicrobial treatment (OAT) were compared across the three study periods. Overall, 134, 112 and 154 positive BC episodes in P0, P1 and P2 respectively were included in the analysis. Mean time to ID (28.3 hours in P0) was reduced by 65.3% in P1 (10.2 hours) and 61.8% in P2 (10.8 hours). Mean time to complete susceptibility results was reduced by 27.5% in P1 and 27% in P2, with results obtained after 32.4 and 32.6 hours compared to 44.7 hours in P0. Rapid tests allowed partial susceptibility results to be obtained after a mean time of 11.8 hours in P1 and 11.7 hours in P2. Mean time to OAT was decreased to 21.6 hours in P1 and to 17.9 hours in P2 compared to 36.1 hours in P0. Reducing TAT of positive BC with MALDI-TOF MS ID and rapid susceptibility testing accelerated prescription of targeted antimicrobial treatment thereby potentially improving the patients’ clinical outcome.

## Introduction

Sepsis is a frequent and severe condition associated with high morbidity and mortality rates. According to the US Centers of Disease Control and Prevention, with over 1 million cases of sepsis occurring each year, sepsis is the ninth leading cause of disease-related deaths in the USA [1]. Prevention, rapid accurate diagnostic tests and innovative treatments constitute the key interventions of a multimodal approach aimed to improve sepsis outcome. The clinical laboratory plays a key role in the diagnosis of bloodstream infections (BSI) and reducing time to identification and susceptibility is a major goal. The rapid availability of results should allow early administration of targeted antimicrobial treatment hereby potentially improving clinical outcome and also reducing length of hospital stay and associated costs [2]. More rapid optimal therapy furthermore limits antibiotic use and the development of resistance [3,4].

Matrix-assisted laser desorption ionization time-of-flight mass spectrometry (MALDI-TOF MS) has proven over the years to be a rapid and accurate universal method for the identification of microorganisms [5]. Various studies have shown that the use of MALDI-TOF MS both decreases the time to organism identification and to effective antibiotic therapy [6,7,8,9]. However antimicrobial susceptibility testing is also essential for selecting the optimal treatment. Rapid resistance testing methods have been introduced in clinical microbiology laboratories for the detection of third generation cephalosporin-resistant Enterobacteriaceae (EB) and *Pseudomonas aeruginosa* or for the detection of methicillin resistance in *Staphylococcus aureus* [10,11,12,13]. These partial susceptibility tests have been validated for their diagnostic performance although their clinical impact has not been thoroughly assessed.

At the Cliniques universitaires St Luc—UCL we set up a routine laboratory procedure for positive blood cultures integrating MALDI-TOF MS identification and rapid susceptibility testing allowing earlier result communication (i.e.: on the day of positivity detection) [14]. This modified workflow was conceived so as to be applicable in a routine clinical setting with a major consideration towards cost-benefit ratio and reduced hands-on time.

The objectives of this study were to measure the time impact of a modified laboratory positive blood culture workflow on the availability of identification and susceptibility results as well as on the time to administration of the optimal antimicrobial treatment.

## Materials and Methods

### Study design

This study was conducted at the Cliniques universitaires St Luc—UCL in Brussels, Belgium, a 964-bed tertiary hospital. This intervention study included three distinct study periods between September 2013 and November 2014. A standard workflow was applied during the pre-intervention period (P0) (September 2013 to November 2013). A modified workflow was identically applied during both intervention periods: period 1 (P1) (from February 2014 to April 2014) and period 2 (P2) (from September 2014 to November 2014). In-between the three periods, laboratory technicians were trained on the modified workflow and multiple educational meetings were held with the antimicrobial stewardship team of the hospital. This team included 2 infectious disease physicians and 1 intensive care practitioner.

All episodes of positive blood cultures occurring in adult patients (≥18 years), based on alerts of the BACTEC FX automated system (Becton Dickinson, Franklin Lakes, NJ, USA) and yielding the growth of a single bacteria or yeast validated for identification with MALDI-TOF MS were included. Any positive blood culture with the same pathogen within one week of a previously confirmed blood culture in the same patient was considered as part of the same episode. Microbiological data of the positive blood culture episodes were recorded from the laboratory information system and patients’ medical records were reviewed for the collection of demographic characteristics and for antibiotic treatment administrative data. The classification of bacteremic episodes into BSI or into contaminations was defined according to the US Centers for Disease Control and Prevention/National Healthcare Safety Network definitions of bloodstream infection events [15]. Patients who died or were transferred to another hospital between blood culture sampling and time to detection as well as patients benefiting from palliative care were excluded from the study.

### Identification and susceptibility methods

Identification was performed by MALDI-TOF MS on a MicroFlex LT platform (Bruker Daltonik, Bremen, Germany). The acquired bacterial spectra were analyzed in the MALDI Biotyper 3.0 software with database version 3.1.2 and bearing the spectra of 4,111 cellular organisms. According to the selected workflow (detailed below) and the time of blood culture positivity detection, identification was performed following one of the three protocols namely (1) direct, (ii) early and (iii) subculture MALDI-TOF MS identification (ID). Direct MALDI-TOF MS ID from positive blood culture fluid with the commercial Sepsityper kit (Bruker Daltonik, Bremen, Germany) performed according to Morgenthaler et al. and early MALDI-TOF MS ID on a young subculture (5 hours) as validated by Verroken et al., allowed result reporting on the same day as blood culture positivity detection [14,16]. MALDI-TOF MS ID on an overnight subculture led to a result the day following detection [17]. MALDI-TOF MS identification result scores were interpreted according to former publications that validated each method [14,16,17].

Antimicrobial susceptibility testing (AST) for staphylococci, enterococci and EB was performed using the automated Phoenix system (Becton Dickinson, Franklin Lakes, NJ, USA) requiring 6–8 hours to complete results. AST of all other bacteria (anaerobes, Corynebacteriaceae, non fermenters, streptococci) was performed by disk diffusion using filter paper disks (Bio-Rad, Marnes-la-Coquette, France) and results were read after 18 hours following EUCAST guidelines [18]. According to the selected workflow and the time of blood culture positivity detection, AST was performed either directly from the blood culture fluid according to Beuving et al. either on an early subculture or on an overnight subculture [19]. Molecular testing was done afterwards on all third generation cephalosporin-resistant and carbapenem-resistant EB and *P*. *aeruginosa* for the detection of extended-spectrum β-lactamases (ESBL) and of carbapenemases [20,21].

Partial susceptibility testing performed during the intervention periods, included the βLACTA Test (βLT) (Bio-Rad, Marnes-la-Coquette, France) and the PBP2a Culture Colony Test (PBP2a) (Alere, Scarborough, ME, USA). βLT was performed from positive blood cultures identified with EB (excluding chromosomal AmpC producers) and *P*. *aeruginosa* leading to the detection of resistance to third generation cephalosporins within 30 minutes. PBP2a was performed on *S*. *aureus* identified blood cultures allowing the detection of methicillin-resistant *S*. *aureus* (MRSA) strains within 15 minutes. According to the time of blood culture positivity detection, partial susceptibility testing was performed directly from blood (on the bacterial pellet obtained following centrifugation of a separator gel tube), or alternatively from early or overnight subcultures following the manufacturer’s instructions.

### Positive blood culture standard and modified laboratory workflow

The microbiology laboratory runs 7days/7 from 8 AM to 6 PM with a more limited activity (Gram staining and inoculation of positive blood cultures only) between 6 PM and 0 AM. The incubation of blood cultures is performed at any time of the day every day (i.e. 24/7).

**Fig 1:**
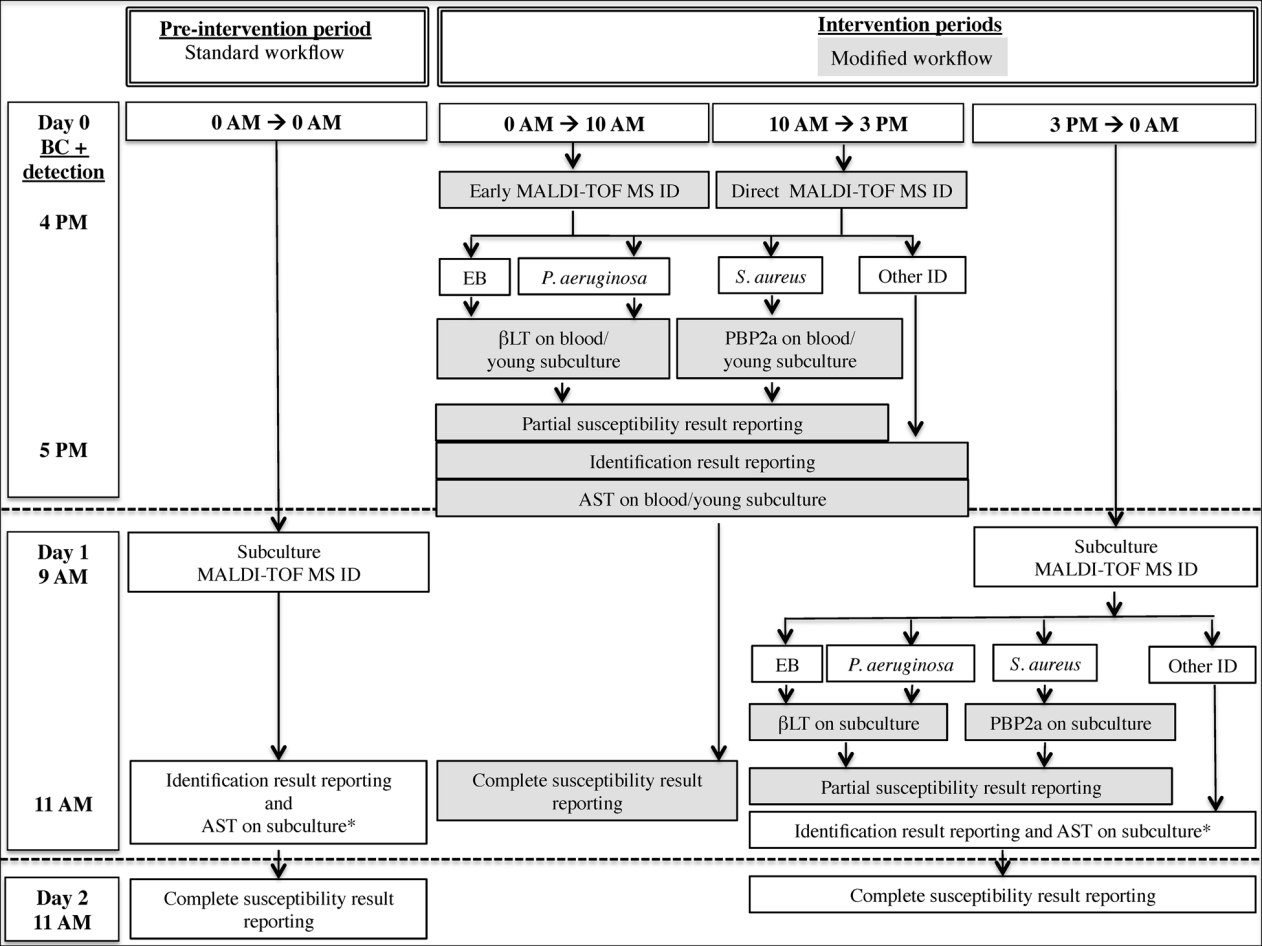
Laboratory workflow of positive blood culture bottles according to time of positivity detection during pre-intervention and intervention periods. AST, antimicrobial susceptibility testing; BC, blood culture; βLT, βLACTA test; EB, Enterobacteriaceae; ID, identification; MALDI-TOF MS, matrix-assisted laser desorption ionization time-of-flight mass spectrometry; PBP2a, PBP2a Culture Colony Test. *: AST was directly performed from positive blood culture fluid when Gram staining identified a Gram-negative rod. Grey squares highlight the accelerated identification and susceptibility tests applied in the modified workflow.

During all three study periods, Gram staining was similarly performed at the time of blood culture positivity detection and immediately communicated by phone to the treating physicians (except between 0 AM and 8 AM). The laboratory workflow of the positive blood culture bottles according to their time of positivity detection during P0, P1 and P2 is detailed in Fig 1.

During phase P0, the standard workflow included subculture MALDI-TOF MS ID on day 1 followed by AST with results available on day 2 of positive blood culture detection. A direct AST by Phoenix automate was performed when Gram-negative rods were detected on Gram staining and susceptibility results were made available on day 1 if an EB was identified.

During the intervention periods (P1 and P2), all blood cultures that were found positive before 10AM on day 0 benefited from the modified workflow that included early subculture MALDI-TOF MS ID, selectively followed by partial susceptibility testing realized from early subculture. For blood cultures detected positive between 10 AM and 3 PM, MALDI-TOF MS ID and partial susceptibility testing was directly performed on blood. ID results were available by 5 PM on the same day. Subsequent AST performed on blood/early subculture allowed complete susceptibility results to be accessible on day 1. Because of limited laboratory staff resources during evening and night hours, blood cultures detected positive after 3 PM were handled using the standard workflow for identification and complete AST. Partial susceptibility testing on subcultured EB, *P*. *aeruginosa* and *S*. *aureus* was performed on day 1 after identification results’ availability, further finalized by complete AST results obtained on day 2.

Twice daily, at 11 AM and 5 PM, all newly available identification, partial or complete susceptibility results were notified by phone to the antimicrobial stewardship team without distinction of the study periods. Recommendations for (semi-) empirical and final targeted antibiotic treatment provided to the prescribing physicians by the antimicrobial stewardship team were based on local antibiotic guidelines and remained similar across all study phases.

### Outcomes

The study objectives were to analyze microbiological and clinical outcomes following implementation of the modified laboratory workflow applied on positive blood cultures. In order for analyses to be comparable between phases, we excluded from the analysis any blood culture from P0 that turned positive at a time of the day that would have made them ineligible for the modified workflow if it had been applied at that time. Similarly, only the positive blood cultures that benefited from the modified blood culture workflow during P1 and P2 were included in the final analysis. Main microbiological outcome indicators were time to identification and time to partial/ complete susceptibility results across the three study periods. Main clinical outcome indicator was time to patient’s administration of the optimal antimicrobial BSI treatment (OAT) in the three periods. The OAT was defined as the final and targeted drug prescribed by the treating physician following the antimicrobial stewardship team’s guidelines. Each OAT was labeled as either the initiation of an antimicrobial treatment, a de-escalation of the (semi-) empirical treatment, a spectrum broadening of the (semi-) empirical treatment or a switch of regimen of the (semi-) empirical treatment. The empirical treatment was defined as the antimicrobial drug administered before availability of any laboratory results (Gram stain, identification or susceptibility). The categorization as semi-empirical treatment was based on partial laboratory results (Gram and/or identification and/or partial susceptibility).

All time measurements started from the instant the blood culture bottle was detected positive by the BACTEC FX automated system. BSI episodes where the modified workflow techniques did not yield acceptable results (lack of identification results, uninterpretable/erroneous partial susceptibility testing results) as well as contaminations were excluded from the outcome analyses.

### Data handling and statistical analyses

Descriptive analyses were first performed on our data to obtain summary statistics for the therapeutic and clinical outcome of interests, as well as patient characteristics. Proportions and means of binary and continual variables were compared between P0, P1 and P2 using chi-square tests, t-tests and analysis of variance (ANOVA). We then explored the distribution of time to identification, time to susceptibility and time to OAT as a function of intervention phases and other covariates of interests using a negative binomial regression model, which is a Poisson model accounting for overdispersion. Covariates significant at P<0.05 in univariable analysis were considered for multivariable analysis in the model exploring time as a function of phases.

### Ethics statement

The study protocol was approved by the Ethical Committee of the Cliniques universitaires St Luc—UCL (National number: B403). Information from microbiological and clinical files was anonymously analyzed and did not require patients’ informed consent.

## Results

**Fig 2:**
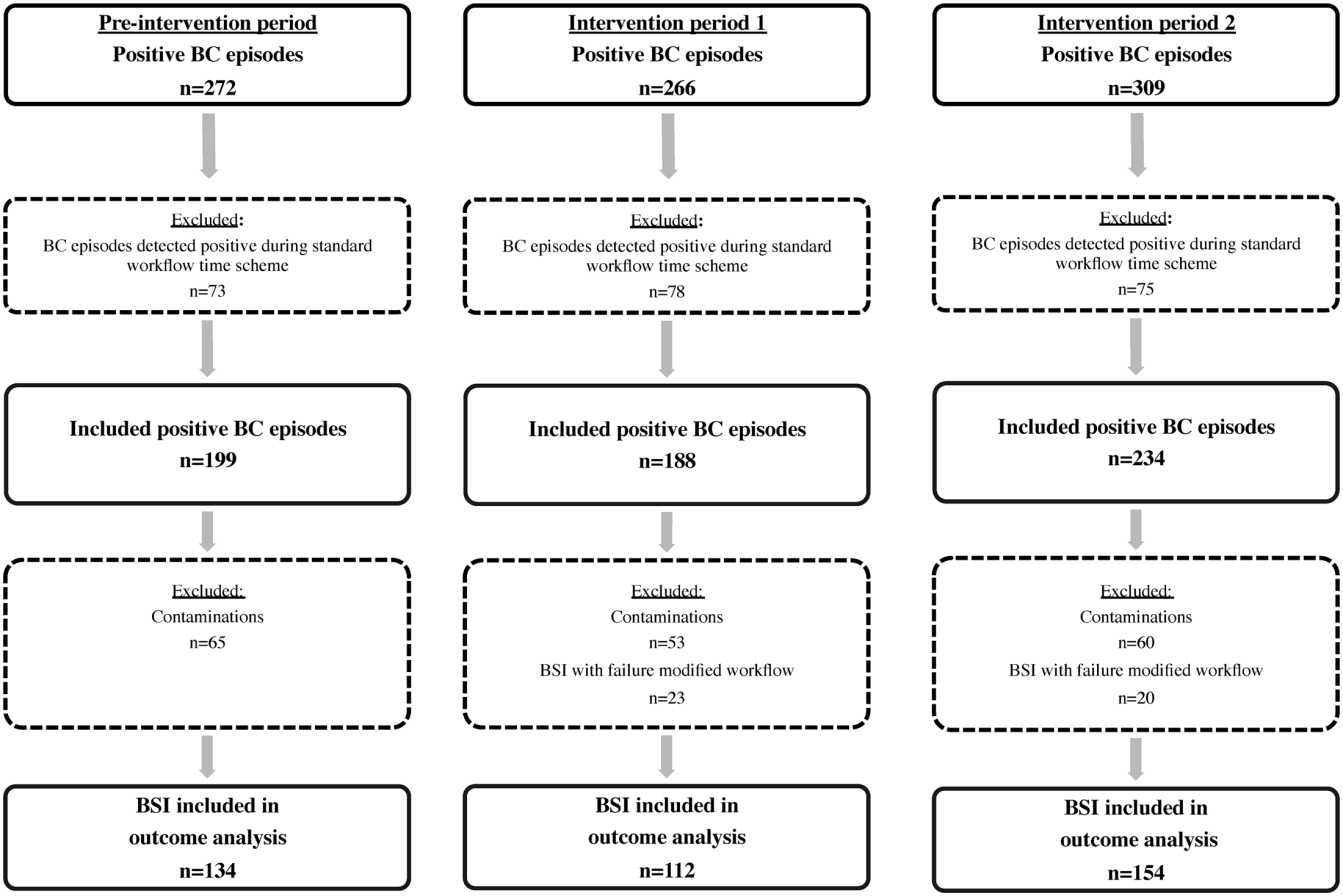
Flowchart of adult monomicrobial blood culture episodes included during pre-intervention and intervention periods. BC, blood culture; BSI, bloodstream infections.

The study included a total of 847 positive blood cultures counting for 272, 266 and 309 episodes during P0, P1 and P2, respectively (Fig 2). The exclusion of all episodes detected positive outside the modified workflow scheme hours, lead to the selection of 199 episodes (73%) in P0, 188 (71%) in P1 and 234 (76%) in P2. With the focus of our study set on BSI, all contaminations were also discarded. Ultimately microbiological and clinical outcomes were compared for 134, 112 and 154 BSI in P0, P1 and P2 respectively as we likewise excluded 23 BSI in P1 and 20 BSI in P2 with unsuccessful results through speeded-up testing.

Demographic characteristics including gender, age, comorbidities selection and source of bacteremia were compared across the three study periods. Overall no statistical differences were observed with an exception for age and diabetes. Patients in P1 and P2 were significantly older and diabetes was more frequent than in P0. In all three periods, the urinary tract, the gastro-intestinal tract and the central venous catheter accounted for the main sources of BSI. Microbiological data and antimicrobial resistance of all BSI are detailed in Table 1. Across the three study periods, non-natural AmpC EB and staphylococci were the most frequent isolated microorganisms. Prevalence of methicillin resistance among *S*. *aureus* was 7.1% in P0, 8.3% in P1 and 14.3% in P2. Prevalence of third generation cephalosporin resistance among EB (natural and non-natural AmpC producers) was 16.9%, 17.6% and 22.6% in P0, P1 and P2 respectively. No carbapenem-resistant EB were isolated in any study period and a single carbapenem-resistant *P*. *aeruginosa* isolate was identified in P1.

**Table 1:** Distribution of microorganisms and main resistances of all bloodstream infections across the three study periods. 3GC, third generation cephalosporin (cefotaxime, ceftriaxone, ceftazidime); AST, antimicrobial susceptibility testing; BSI, bloodstream infection; carbapenem (imipenem, meropenem); ID, identification; P0, pre-intervention period; P1, intervention period 1; P2, intervention period 2. Natural AmpC producers identified during the study periods: *Citrobacter freundii*, *Enterobacter aerogenes*, *Enterobacter cloacae*, *Hafnia alvei*, *Serratia marcescens*. Non-natural AmpC producers identified during the study periods: *Citrobacter koseri*, *Escherichia coli*, *Klebsiella oxytoca*, *Klebsiella pneumoniae*, *Proteus mirabilis*, *Proteus vulgaris*, *Salmonella spp*.

Microorganism			Resistance	P0	P1			P2		
				n BSI (%)	n BSI (%)	n BSI	n BSI	n BSI (%)	n BSI	n BSI
				included in outcome analysis	included in outcome analysis	excluded from outcome analysis		included in outcome analysis	excluded from outcome analysis	
						with failed speeded-up ID	with failed partial AST		with failed speeded-up ID	with failed partial AST
**Gram-positive bacteria**				**50 (37.3)**	**40 (35.7)**	**8**	**0**	**44 (28.6)**	**7**	**0**
	Staphylococci			25	22	3	0	24	2	0
		*Staphylococcus aureus*		14	11	1	0	14	0	0
			methicillin	1	1	0	-	2	-	-
		Coagulase negative staphylococci		11	11	2	0	10	2	0
	Enterococci			12	11	1	0	9	1	0
	Streptococci			9	7	2	0	10	4	0
	Other Gram-positive bacteria			4	0	2	0	1	0	0
**Gram-negative bacteria**				**77 (57.5)**	**71 (63.4)**	**1**	**6**	**107 (69.5)**	**2**	**2**
	Enterobacteriaceae			71	63	0	5	103	1	2
		natural AmpC producers		5	6	0	0	11	0	0
			3GC	2	1	-	-	4	-	-
			carbapenem	0	0	-	-	0	-	-
		non-natural AmpC producers		66	57	0	5	92	1	2
			3GC	10	6	-	5	18	0	2
			carbapenem	0	0	-	0	0	0	0
	Non fermenters			6	6	1	1	4	1	0
		*Pseudomonas aeruginosa*		3	4	0	1	3	1	0
			3GC	0	0	-	1	0	0	-
			carbapenem	0	0	-	1	0	0	-
		Other non fermenters		3	2	1	0	-	0	0
	Other Gram-negative bacteria			0	2	-	0	1	0	0
**Anaerobes**				**6 (4.5)**	**0 (0.0)**	**6**	**0**	**2 (1.3)**	**6**	**0**
**Yeast**				**1 (0.7)**	**1 (0.9)**	**2**	**0**	**1 (0.6)**	**3**	**0**
**TOTAL**				**134 (100)**	**112 (100)**	**17**	**6**	**154 (100)**	**18**	**2**

### Rapid identification and susceptibility test performances

Failure of identification with direct/early MALDI-TOF MS led to the exclusion of respectively 17 and 18 BSI in P1 and P2 (Table 1). Insufficient log scores or the absence of spectral peaks were at the origin of all missed identifications. Anaerobes and yeast were the main non-identified microorganisms. There were no misidentifications. In conclusion, considering all BSI managed with the modified workflow in both intervention periods, direct and early MALDI-TOF MS allowed a reliable species identification for 82.0% and 87.0% episodes, respectively.

βLT performed on 165 BSI (P1 and P2 combined) yielded 24 positive, 140 negative and 1 uninterpretable test result due to an incoherent color change of the chromogenic test. All positive βLT results were found in non-natural AmpC EB isolates displaying third generation cephalosporin resistance by complete AST results and subsequently confirmed as ESBL producers. In 7 cases, βLT yielded false-negative results since complete AST and molecular testing identified 6 AmpC producing *Escherichia coli* and 1 VIM metallo-beta-lactamase-producing *P*. *aeruginosa*, all resistant to third generation cephalosporin. Globally, sensitivity and specificity of βLT were respectively 77.4% and 100%. No erroneous or uninterpretable results were observed with PBP2a testing. Performed on 26 *S*. *aureus* BSI in P1 and P2, PBP2a was able to detect all 3 MRSA strains (sensitivity and specificity of 100%). Ultimately, 8 BSI (6 in P1 and 2 in P2) were discarded from outcome analysis due to erroneous/uninterpretable rapid test results.

### Microbiological outcomes

**Fig 3:**
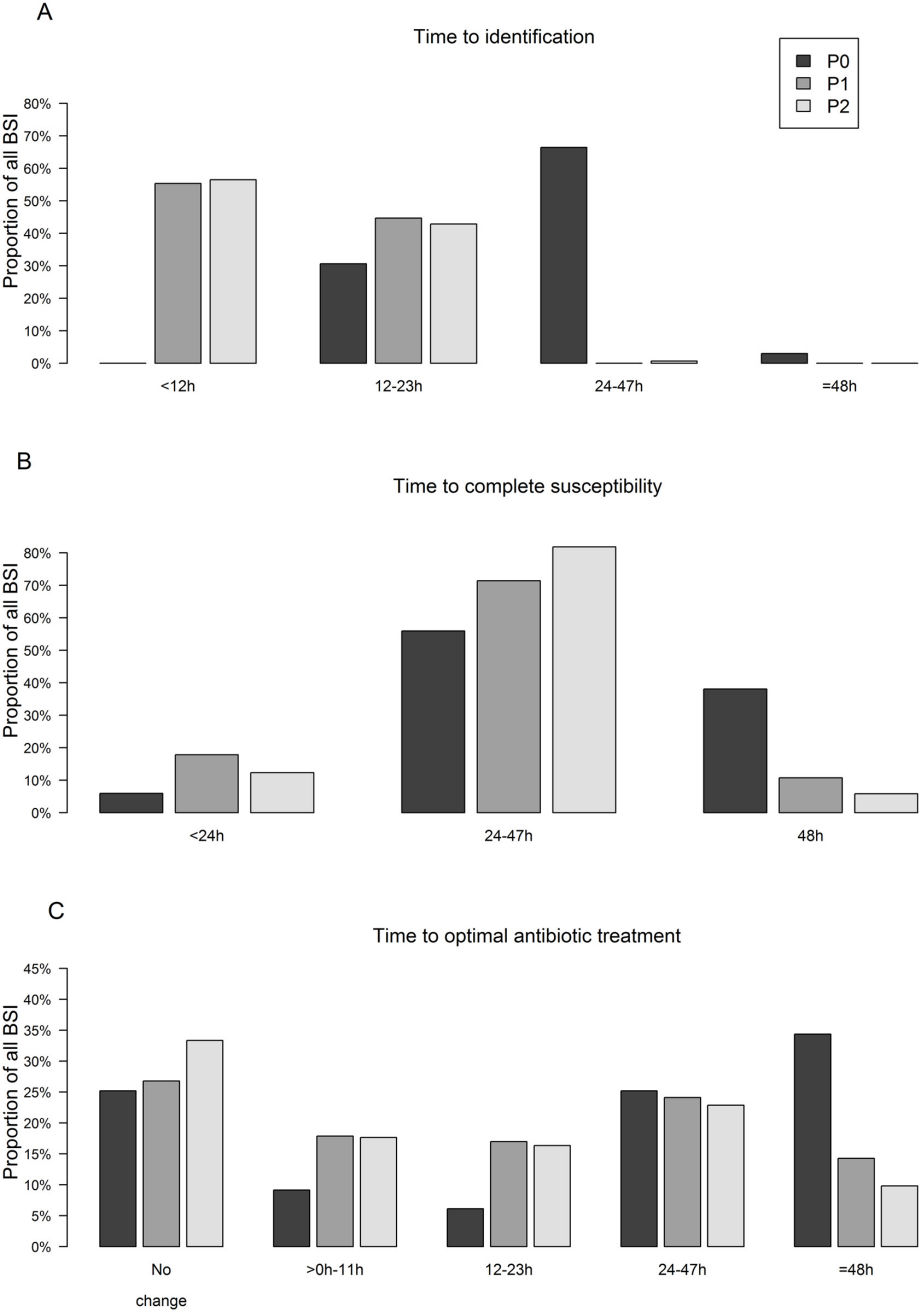
Distribution of the bloodstream infections over time in the three study periods according to their microbiological and clinical outcomes. (A) Distribution of the bloodstream infections identified within defined time lapses. (B) Distribution of the bloodstream infections according to the time of complete susceptibility result availability. (C) Distribution of the bloodstream infections benefitting from the optimal antimicrobial treatment within defined time lapses. The “No change” category defines bloodstream infections optimally treated before the detection of positive blood culture. BSI, bloodstream infection.

Microbiological outcomes are presented in Table 2. Time to identification of positive blood culture episodes was significantly reduced in P1 and P2 compared to P0. The mean time was 28.3 hours in P0 and was reduced by 65.3% in P1 (10.2 hours) and 61.8% in P2 (10.8 hours) (P<0.0001). In P1, subculture, early and direct MALDI-TOF MS were respectively performed on 23/112 (20.5%), 65/112 (58.0%) and 24/112 (21.5%) blood cultures. During P2, 32/154 (20.8%) subculture, 96/154 (62.3%) early and 26/154 (16.9%) direct MALDI-TOF MS identifications were realized. Combining P1 and P2, mean time to identification when applying subculture, early and direct MALDI-TOF MS ID protocols were respectively 16.5, 10.7 and 3.8 hours. Fig 3A shows the distribution of BSI identified within 12, 24 and 48 hours for the 3 periods and highlights the higher proportion of BSI identified within 12 and 24 hours in P1 and P2 compared to P0. As for identification, the mean time to complete susceptibility results was reduced by 27.5% in P1 and 27% in P2 with results after 32.4 and 32.6 hours compared to 44.7 hours in P0 (P<0.0001). Fig 3B highlights the reduced time to complete susceptibility in P1 and P2 compared to P0. The larger proportion of BSI with complete AST results within 48 hours in P1 and P2 could be assigned to the extension of direct AST to staphylococci and enterococci and the integration of manual/automated testing on young subcultures as detailed in Table 2. Rapid βLT and PBP2a allowed partial susceptibility results after a mean time of 11.8 hours in P1 and after 11.7 hours in P2.

**Table 2:** Time to identification and time to partial/complete susceptibility results of all bloodstream infections during pre-intervention and intervention period 1 and 2.

**Time to identification**			
Phase	Method	n BSI	Mean time to ID (hours)
**P0**	**TOTAL**	**134**	**28.3**
	Subculture MALDI-TOF MS	134	28.3
	Early MALDI-TOF MS	-	-
	Direct MALDI-TOF MS	-	-
**P1**	**TOTAL**	**112**	**10.2**
	Subculture MALDI-TOF MS	23	15.9
	Early MALDI-TOF MS	65	10.6
	Direct MALDI-TOF MS	24	3.6
**P2**	**TOTAL**	**154**	**10.8**
	Subculture MALDI-TOF MS	32	17.1
	Early MALDI-TOF MS	96	10.7
	Direct MALDI-TOF MS	26	4.0
**Time to partial susceptibility result**			
Phase	Method	n BSI	Mean time to partial AST result (hours)
**P1**	**TOTAL**	**72**	**11.8**
	Culture βLT	20	17.9
	Young subculture βLT	29	11.2
	Direct βLT	12	3.7
	Culture PBP2a	3	15.3
	Young subculture PBP2a	6	12
	Direct PBP2a	2	2
**P2**	**TOTAL**	**108**	**11.7**
	Culture βLT	29	16.8
	Young subculture βLT	50	11.1
	Direct βLT	15	3.6
	Culture PBP2a	4	20.2
	Young subculture PBP2a	9	8.8
	Direct PBP2a	1	6
**Time to complete susceptibility result**			
Phase	Method	n BSI	Mean time to complete AST result (hours)
**P0**	**TOTAL**	**134**	**44.7**
	Phoenix from subculture	58	46.9
	Phoenix from young subculture	-	-
	Direct Phoenix	48	28.3
	Manual testing from subculture	28	68.3
	Manual testing from young subculture	-	-
**P1**	**TOTAL**	**112**	**32.4**
	Phoenix from subculture	28	41.2
	Phoenix from young subculture	23	32.2
	Direct Phoenix	45	25.6
	Manual testing from subculture	7	49.1
	Manual testing from young subculture	9	27.3
**P2**	**TOTAL**	**154**	**32.6**
	Phoenix from subculture	34	41.6
	Phoenix from young subculture	81	29.9
	Direct Phoenix	22	22.2
	Manual testing from subculture	8	52.2
	Manual testing from young subculture	9	30.9

AST, antimicrobial susceptibility testing; βLT, βLACTA test; BSI, bloodstream infection; ID, identification; MALDI-TOF MS, matrix-assisted laser desorption ionization time-of-flight mass spectrometry; P0, pre-intervention period; P1, intervention period 1; P2, intervention period 2; PBP2a, PBP2a Culture Colony Test.

### Clinical outcomes

Time to administration of the OAT was significantly reduced during the intervention periods with mean times of 21.6 hours in P1 and 17.9 hours in P2 compared to 36.1 hours in P0 (P<0.0001). Fig 3C displays the higher proportion of BSI optimally treated within the first 24 hours in P1 and P2 versus P0. The faster identification and susceptibility test results of the modified workflow in P1 would have allowed earlier initiation of a targeted antimicrobial treatment in 43/112 BSI episodes (38.4%) yet the antimicrobial stewardship team considered laboratory results and consequently adapted therapy in only 22 BSI (19.6%). Similarly in P2, 64/154 BSI (41.6%) episodes had speeded-up laboratory results theoretically allowing the accelerated onset of the OAT yet an effective therapy switch was introduced only in 45 BSI episodes (29.2%). Table 3 illustrates all BSI episodes from P1 and P2 that benefited from an effective speeded-up antimicrobial treatment tailoring following rapid laboratory test results. Early and direct MALDI-TOF MS identification majorly contributed to the earlier initiation of antibiotic therapy in BSI episodes without empirical treatment. All strains identified in these 18 BSI episodes were Gram-positive microorganisms. Concomitant PBP2a test results assisted to the early onset of the OAT in 4 methicillin-susceptible (1 in P1 and 3 in P2) and 2 methicillin-resistant *S*. *aureus* BSI (1 in P1 and 1 in P2). In BSI with an inappropriate empirical treatment, rapid identification and partial susceptibility testing results equally facilitated treatment tailoring. Thereby 13 positive βLT results (5 in P1 and 7 in P2) led to the accelerated OAT administration through antimicrobial spectrum broadening or a switch to a different antimicrobial regimen. Conversely, 14 negative ßLT and PBP2a results (1 in P1 and 13 in P2) allowed faster treatment de-escalation in BSI with an appropriate empirical BSI treatment. For 69 BSI in P1 and 90 BSI in P2, the laboratory results inherent to the modified workflow brought no additional information enabling the tailoring of the administered empirical antibiotic treatment. Those episodes included inter alia all BSI with an empirical treatment remaining unchanged because ultimately evaluated as the OAT (“No change” category in Fig 3C).

**Table 3:** Effective antibiotic treatment tailoring of bloodstream infections in both intervention periods following accelerated laboratory results.

	BSI without empirical treatment		BSI with inappropriate empirical treatment		BSI with appropriate empirical treatment	
Intervention period (n BSI)	P1 (10)	P2(8)	P1 (8)	P2 (16)	P1 (4)	P2 (21)
**Subsequent antimicrobial treatment tailoring**						
Initiation	10	8	-	-	-	-
De-escalation	-	-	-	-	3	17
Spectrum broadening	-	-	4	13	-	-
Switch of regimen	-	-	4	3	1	4
**Speeded-up test at the origin of treatment tailoring**						
Direct/early MALDI-TOF MS ID	10	8	3	7	2	4
Rapid partial susceptibility testing						
PBP2a	2*	4*	-	-	1	1
βLT	-	-	5	7	-	12
Direct/young subculture complete AST	-	-	-	2	1	4

AST, antimicrobial susceptibiltiy testing; βLT, βLACTA test; BSI, bloodstream infection; ID, identification; MALDI-TOF MS, matrix-assisted laser desorption ionization time-of-flight mass spectrometry; P1, intervention period 1; P2, intervention period 2; PBP2a, PBP2a Culture Colony Test.

*The rapid partial susceptibility test result contributed to a treatment tailoring in combination with the identification test result.

## Discussion

The modified workflow evaluated in our study included MALDI-TOF MS identification, rapid partial susceptibility tests (PBP2a and ßLT) for *S*. *aureus*, EB and *P*. *aeruginosa*, and manual/automated complete susceptibility testing. Additionally, all tests could be carried out from three different supports (blood culture pellets, early subcultures and overnight subcultures) depending on the timeframe during which the blood culture bottle was detected positive. Through the set-up of this workflow, we aimed to maximize the proportion of microbiological results made available to the clinicians within the same day of positive blood culture detection in balance with hands-on time of tests, technician availability and reagent costs. All blood cultures detected positive before 10 AM yielded sufficient microbial growth for performing the tests directly from subculture colonies in the early afternoon. The high performances of early MALDI-TOF MS with 87% correct identification and it’s ease of use, requiring no fastidious pre-treatment nor any additional cost, makes it an excellent alternative in comparison to direct MALDI-TOF MS [22]. On the other hand, blood cultures detected after 10 AM did not provide sufficient growth in the early afternoon for rapid testing and thus required direct identification. With reliable results obtained in 82%, we achieved the global 80% performance of the Sepsityper kit calculated in the meta-analysis of Morgenthaler et al. [16]. Blood cultures detected positive between 3 PM and 0 AM were identified by classical subculture MALDI-TOF MS on the following day. This is due to the fact that our microbiology laboratory is not fully operational on a 24-hour basis as only limited work is realized from ongoing analyses during the late afternoon and night. Eveillard et al. demonstrated the usefulness of performing bacterial identification and AST 24h a day with an earlier initiation of appropriate treatment in 22.6% [23]. But in this current era of global healthcare cost savings, extending laboratory working staff is unlikely and the availability on site of a clinician during late-day and night hours that would adjust antimicrobial treatment according early laboratory results also seems very unlikely. We therefore believe that the proposed identification workflow may constitute a good alternative as speeded-up MALDI-TOF MS enabled identification results within 24 hours after positive blood culture detection for > 95% of all BSI during the two intervention periods.

Alongside rapid partial susceptibility testing likewise provided contributive results on the day of blood culture positivity detection. Despite the limited information, a partial susceptibility test result can give some valuable insight and help predicting the antibiotic phenotypic resistance profile of an organism. Such information, when coupled with the knowledge of local resistance epidemiology, can improve the prescription of targeted antibiotic treatment. PBP2a testing yielded excellent performances as it detected all MRSA blood strains without any false positive results being found. Additionally, 2 positive PBP2a results led to the earlier instauration of containment measures following the detection of a multi-resistant strain. On the other hand, βLT performances are more nuanced as the test missed the detection of 7 third generation cephalosporin-resistant strains i.e. 6 AmpC producing *E*. *coli* and one VIM *P*. *aeruginosa*. Previously, Walewski et al. underlined the low sensitivity of ßLT for the detection of chromosomal overproduced AmpC-type ß-lactamases but also of plasmid-encoded AmpC [13]. We believe additional validation and improvement of the ßLT is necessary; until then caution needs to be taken in the treatment adaptation of BSI episodes caused by non-natural AmpC producing EB with a negative ßLT. The inability of ßLT to detect a VIM-producing *P*. *aeruginosa* supported earlier observations made by Laurent et al. [11].

Our study approach distinguishes itself as it measures the clinical impact of a complete positive blood culture workflow rather than the isolated impact of a single identification or susceptibility test. Several authors evaluated the impact of MALDI-TOF MS identification on antimicrobial prescription in patients with bacteremia [6,7,8,9]. In the prospective trial of Vlek et al., direct MALDI-TOF MS performed twice a day led to a median time to identification of 16.4 hours and was associated with 64% of the patients receiving appropriate treatment 24 hours after blood culture positivity [9]. Our study was similarly conducted but contained additional rapid identification and susceptibility techniques thereby reducing mean time to OAT from 31.1 hours to 21.6 and 17.9 hours respectively in P1 and P2. Early and direct MALDI-TOF MS identification mainly contributed to earlier initiation of antibiotic therapy in BSI episodes that had previously been considered as contaminations especially in non-critically ill patients. Partial susceptibility test results led more often to the adjustment of an already administered antimicrobial therapy by streamlining or broadening the spectrum. Interestingly, we observed a better empiric coverage during P2 versus P1 and P0 potentially influencing the reduced time to OAT. This observation suggests that besides the effect of the modified workflow, other factors as for example an increasing consideration of rapid laboratory test results over time by the antimicrobial stewardship team and a better adhesion to empiric treatment guidelines might also have played a role in our study.

The division of the intervention phase into two sequential periods allowed us to evaluate the clinician’s prescription behavior over time. Of all BSI with speeded-up microbiological results enabling an accelerated prescription of OAT, 70.3% (45/64) benefited from an effective treatment revision in P2 compared to 51% (22/43) in P1. Educational meetings between the two intervention periods discussing rapid testing performances and partial outcome results possibly could have led to an enhanced trust of the infection disease specialists and thereby might have accounted for the improved time to OAT in P2 versus P1.

The modified workflow for the management of positive blood cultures outlined in the study is a highly customized scheme adapted to our local working environment and in accordance with our local resistance epidemiology. The transposition of this scheme in another setting could have a different impact on the clinical outcomes depending on local microbiological and resistance data and also influenced by the local antimicrobial treatment guidelines for septicemia. Beyond the proposal of an improved yet singular positive blood culture laboratory workflow, this study ultimately aims at arousing the interest of each clinical laboratory to reassess positive blood culture management allowing speeded-up identification and susceptibility results. Our workflow evaluated a non-exhaustive selection of identification and susceptibility testing methods and can only be considered as a primary draft to be refined by each laboratory according to specific local criteria. The selection of an identification method primarily depends on the laboratory working hours. Alongside, rapid susceptibility tests should be selected based on the local resistant epidemiology. In settings with high prevalence/incidence of carbapenem resistance, laboratories should consider the introduction of a distinct rapid partial susceptibility test for the detection of carbapenemases [24]. On the other hand, partial susceptibility tests might have less impact in a clinical setting with very low resistance profiles.

In this study, we aimed to develop a refined applicable positive blood culture workflow integrating rapid identification and susceptibility techniques hereby allowing an accelerated microbial diagnosis and a reduced time to OAT. This integrated approach with the active cooperation of the antimicrobial stewardship team significantly improved the timeliness of targeted treatment administration. Further work evaluating the potential impact of the modified workflow on mortality and morbidity, length of hospitalization and medical costs is suggested.
