# Correction: Clinical Impact of MALDI-TOF MS Identification and Rapid Susceptibility Testing on Adequate Antimicrobial Treatment in Sepsis with Positive Blood Cultures

**DOI:** 10.1371/journal.pone.0160537

**Published:** 2016-09-22

**Authors:** Alexia Verroken, Lydwine Defourny, Olivier le Polain de Waroux, Leïla Belkhir, Pierre-François Laterre, Michel Delmée, Youri Glupczynski

There are numerical errors in the first and penultimate sentences of the second paragraph of the Results under the subheading “Rapid identification and susceptibility test performances.” The correct paragraph is: βLT performed on 164 BSI (P1 and P2 combined) yielded 24 positive, 139 negative and 1 uninterpretable test result due to an incoherent color change of the chromogenic test. All positive βLT results were found in non-natural AmpC EB isolates displaying third generation cephalosporin resistance by complete AST results and subsequently confirmed as ESBL producers. In 7 cases, βLT yielded false-negative results since complete AST and molecular testing identified 6 AmpC producing *Escherichia coli* and 1 VIM metallo-beta-lactamase-producing *P*. *aeruginosa*, all resistant to third generation cephalosporin. Globally, sensitivity and specificity of βLT were respectively 77.4% and 100%. No erroneous or uninterpretable results were observed with PBP2a testing. Performed on 25 *S*. *aureus* BSI in P1 and P2, PBP2a was able to detect all 3 MRSA strains (sensitivity and specificity of 100%). Ultimately, 8 BSI (6 in P1 and 2 in P2) were discarded from outcome analysis due to erroneous/uninterpretable rapid test results.

Tables 1 and 2 appear incorrectly in the published article. Please see the correct Tables 1 and 2 and their captions here.

**Table 1:** Distribution of microorganisms and main resistances of all bloodstream infections across the three study periods. 3GC, third generation cephalosporin (cefotaxime, ceftriaxone, ceftazidime); AST, antimicrobial susceptibility testing; BSI, bloodstream infection; carbapenem (imipenem, meropenem); ID, identification; P0, pre-intervention period; P1, intervention period 1; P2, intervention period 2. Natural AmpC producers identified during the study periods: *Citrobacter freundii*, *Enterobacter aerogenes*, *Enterobacter cloacae*, *Hafnia alvei*, *Serratia marcescens*. Non-natural AmpC producers identified during the study periods: *Citrobacter koseri*, *Escherichia coli*, *Klebsiella oxytoca*, *Klebsiella pneumoniae*, *Proteus mirabilis*, *Proteus vulgaris*, *Salmonella spp*.

Microorganism Resistance	P0	P1			P2		
	n BSI (%)	n BSI (%)	n BSI	n BSI	n BSI (%)	n BSI	n BSI
	final outcome analysis	final outcome analysis	with failed ID	with failed partial AST	final outcome analysis	with failed ID	with failed partial AST
**Gram-positive bacteria**	**50 (37.3)**	**40 (35.7)**	**8**	**0**	**44 (28.6)**	**7**	**0**
*Staphylococci*	25	22	3	0	24	2	0
*Staphylococcus aureus*	14	11	1	0	14	0	0
methicillin	1	1	0	-	2	-	-
Coagulase negative *Staphylococci*	11	11	2	0	10	2	0
*Enterococci*	12	11	1	0	9	1	0
*Streptococci*	9	7	2	0	10	4	0
Other Gram-positive bacteria	4	0	2	0	1	0	0
**Gram-negative bacteria**	**77 (57.5)**	**71 (63.4)**	**1**	**6**	**107 (69.5)**	**2**	**2**
Enterobacteriaceae	71	63	0	5	103	1	2
natural AmpC producers	5	6	0	0	11	0	0
3GC	2	1	-	-	4	-	-
carbapenem	0	0	-	-	0	-	-
non-natural AmpC producers	66	57	0	5	92	1	2
3GC	10	6	-	5	18	0	2
carbapenem	0	0	-	0	0	0	0
Non fermenters	6	6	1	1	4	1	0
*Pseudomonas aeruginosa*	3	4	0	1	3	1	0
3GC	0	0	-	1	0	0	-
carbapenem	0	0	-	1	0	0	-
Other non fermenters	3	2	1	0	**1**	0	0
Other Gram-negative bacteria	0	2	-	0	**0**	0	0
**Anaerobes**	**6 (4.5)**	**0 (0.0)**	**6**	**0**	**2 (1.3)**	**6**	**0**
**Yeast**	**1 (0.7)**	**1 (0.9)**	**2**	**0**	**1 (0.6)**	**3**	**0**
**TOTAL**	**134 (100)**	**112 (100)**	**17**	**6**	**154 (100)**	**18**	**2**

**Table 2:** Time to identification and time to partial/complete susceptibility results of all bloodstream infections during pre-intervention and intervention period 1 and 2.

Time to identification			
Phase	Method	BSI (n)	Mean time to ID (hours)
**P0**	**TOTAL**	**134**	**28.3**
	Subculture MALDI-TOF MS	134	28.3
	Early MALDI-TOF MS	-	-
	Direct MALDI-TOF MS	-	-
**P1**	**TOTAL**	**112**	**10.2**
	Subculture MALDI-TOF MS	23	15.9
	Early MALDI-TOF MS	65	10.6
	Direct MALDI-TOF MS	24	3.6
**P2**	**TOTAL**	**154**	**10.8**
	Subculture MALDI-TOF MS	32	17.1
	Early MALDI-TOF MS	96	10.7
	Direct MALDI-TOF MS	26	4.0
**Time to complete susceptibility result**			
Phase	Method	BSI (n)	Mean time to complete AST result (hours)
**P0**	**TOTAL**	**134**	**44.7**
	Phoenix from subculture	58	46.9
	Phoenix from young subculture	-	-
	Direct Phoenix	48	28.3
	Manual testing from subculture	28	68.3
	Manuel testing from young subculture	-	-
**P1**	**TOTAL**	**112**	**32.4**
	Phoenix from subculture	28	41.2
	Phoenix from young subculture	23	32.2
	Direct Phoenix	45	25.6
	Manual testing from subculture	7	49.1
	Manuel testing from young subculture	9	27.3
**P2**	**TOTAL**	**154**	**32.6**
	Phoenix from subculture	34	41.6
	Phoenix from young subculture	81	29.9
	Direct Phoenix	22	22.2
	Manual testing from subculture	8	52.2
	Manuel testing from young subculture	9	30.9
**Time to partial susceptibility result**			
Phase	Method	BSI (n)	Mean time to partial susceptibility testing result (hours)
**P1**	**TOTAL**	**72**	**11.8**
	Culture βLT	20	17.9
	Young subculture βLT	29	11.2
	Direct βLT	12	3.7
	Culture PBP2a	3	15.3
	Young subculture PBP2a	6	12
	Direct PBP2a	2	2
**P2**	**TOTAL**	**109**	**11.7**
	Culture βLT	29	16.8
	Young subculture βLT	51	11.1
	Direct βLT	15	3.6
	Culture PBP2a	4	20.2
	Young subculture PBP2a	9	8.8
	Direct PBP2a	1	6
